# Public Expectations and Needs Related to Type 2 Diabetes Prevention: A Population-Based Cross-Sectional Study in Poland

**DOI:** 10.3389/ijph.2024.1606790

**Published:** 2024-01-23

**Authors:** Justyna Grudziąż-Sękowska, Kuba Sękowski, Jarosław Pinkas, Mateusz Jankowski

**Affiliations:** School of Public Health, Centre of Postgraduate Medical Education, Warsaw, Poland

**Keywords:** prevention, health education, needs assessment, diabetes mellitus, preventive health services

## Abstract

**Objective:** This study aimed to understand the public’s expectations regarding type 2 diabetes prevention and to identify factors associated with willingness to participate in preventive activities among adults in Poland.

**Methods:** A cross-sectional survey was carried out using a computer-assisted web interview (CAWI) on a representative sample of 1,046 adults in Poland. A non-probability quota sampling method was used. A study tool was a self-prepared questionnaire.

**Results:** Most respondents (77.3%) declared willingness to participate in preventive activities. Consultation with a diabetologist (75.1%) or family doctor consultation (74.9%) were the most often selected. Lifestyle interventions in the form of dietary and culinary workshops (58.1%) were the least chosen. Having higher education (OR = 3.83, 1.64–8.94, *p* = 0.002), chronic diseases (OR = 1.36, 1.01–1.85, *p* = 0.04), and a history of diabetes in the family (OR = 1.67, 1.21–2.30, *p* = 0.002) were significantly associated with a higher interest in type 2 diabetes prevention.

**Conclusion:** The adults in Poland are keen on participating in diabetes prevention programs, mostly those based on medical counselling rather than lifestyle-oriented interventions. Educational level was the most important factor associated with willingness to participate in type 2 diabetes prevention.

## Introduction

Type 2 diabetes, a prevalent chronic disease, imposes a substantial burden on individuals and societies worldwide, resulting in multifaceted health, social, and economic costs. The prevalence of type 2 diabetes has reached pandemic proportions, with an estimated global prevalence of 9.3% in 2019, affecting over 463 million people, and is projected to rise to 10.9% by 2045 [1]. The health-related consequences of type 2 diabetes encompass a range of complications, including cardiovascular disease, neuropathy, nephropathy, and retinopathy, all of which contribute to reduced quality of life and increased mortality rates [1].

Beyond the direct health impact, type 2 diabetes exerts substantial social and economic costs [2, 3]. It necessitates ongoing medical care, including medication and monitoring, straining healthcare systems and increasing healthcare expenditures [2]. Furthermore, individuals with type 2 diabetes often experience reduced productivity and missed workdays due to illness and medical appointments, affecting their economic wellbeing and placing a financial burden on society [3].

The preventable nature of type 2 diabetes underscores the pivotal role of patient involvement in effective diabetes prevention strategies [1, 4]. Recent research has demonstrated that lifestyle modifications, including dietary improvements, increased physical activity, and weight management, can significantly reduce the risk of developing type 2 diabetes among high-risk individuals [4, 5]. These interventions necessitate active patient engagement, demanding sustained behavioral changes and adherence to health-promoting practices. Patient involvement extends beyond mere compliance with medical recommendations; it encompasses education, empowerment, and self-management skills, all of which are integral to the success of diabetes prevention efforts [6]. Encouraging individuals to take an active role in their health and providing them with the necessary tools and support to make informed decisions and sustain healthy lifestyles are paramount in the fight against type 2 diabetes.

There is an increasing recognition of the need to take measures to reduce the risk, detect early, and limit the consequences of type 2 diabetes. Diabetes prevention has become a priority at the global [7], regional [8], and national levels.

In Poland, central and local authorities are taking action to prevent diabetes under The National Health Programme 2021–2025 [9]. Nonetheless, type 2 diabetes prevalence in Poland is on the rise [10]. It is estimated that the number of patients diagnosed with diabetes exceeds 2.5 million [11], and up to a million are unaware of the diagnosis [12].

Preventing type 2 diabetes involves two main strategies: primary and secondary prevention. Primary prevention aims to reduce the occurrence and progression of the condition in individuals without diabetes who are at risk due to factors such as obesity, physical inactivity, and poor dietary habits [13]. Lifestyle modification programs are crucial in primary prevention, focusing on behavioural changes like healthy eating, regular exercise, and weight management to lower the risk of developing type 2 diabetes [14]. Secondary prevention involves early detection of the disease in seemingly healthy individuals who have subclinical forms of diabetes. Secondary prevention strategies often involve targeted [15] or opportunistic [16] screening (mainly blood sugar testing) and advice or counselling provided by medical professionals (i.e., brief intervention) [17].

Barriers to effective preventive actions include organizational issues such as administrative or financial limitations [18, 19], as well as participants’ dependent factors that limit attendance in offered activities [20, 21]. The importance of determining the scope/method of intervention following participant expectations and capabilities [22] and selecting the appropriate outreach [23] strategies to reach the proper target population was underlined.

Therefore, the objective of this study was to characterize public expectations and needs relating to type 2 diabetes prevention and identify factors associated with willingness to participate in activities aimed at type 2 diabetes prevention among adults in Poland as determinants of the effectiveness of preventive measures.

## Methods

### Study Design and Sample

This cross-sectional study was conducted between 15 and 18 September 2023 on a representative sample of 1,046 adults in Poland.

Data were collected using a computer-assisted web interview (CAWI) technique by the professional public opinion research company (Nationwide Research Panel Ariadna, Warsaw, Poland), which acted on behalf of the research team [24]. The participants in the survey were chosen from a pool of over 100,000 registered and verified individuals who actively participate in web-based surveys conducted by the public opinion research company [24]. A non-probability quota sampling method was used, with a stratification model that accounted for variables such as gender, age, size, and location of the place of residence. This stratification was based on sociodemographic datasets collected and published by the Central Statistical Office of the Republic of Poland in Warsaw. Similar methods were used in previous population-based cross-sectional studies in Poland [25, 26].

This study was approved by the Ethical Review Board at the Centre of Postgraduate Medical Education, decision number 404/2023 as of 23 August 2023.

### Participants and Public Involvement

Participants in this study were not involved in developing the design or recruitment. Results will be disseminated via publication in an open-access journal.

### Measures

The questionnaire was self-prepared and based on a literature review [15–20]. The study questionnaire included ten questions on public health interventions related to type 2 diabetes prevention. Additionally, questions on sociodemographic characteristics were addressed. A pilot survey was carried out. A group of 11 adults (aged from 19 to 73 years) filled out the questionnaire twice, 7 days apart. Responses from the pilot survey were analyzed and two questions (including answer options) were modified to clarify the text.

Willingness to participate in activities aimed at type 2 diabetes prevention: Respondents were asked about their willingness to participate in activities aimed at type 2 diabetes prevention, using the question: „Would you like to take advantage of activities aimed at preventing type 2 diabetes?” with a 5-point Likert scale.

Public interest in various activities aimed at type 2 diabetes prevention: Respondents were asked about their interest in various activities aimed at type 2 diabetes prevention, using the question: “Which activities aimed at type 2 diabetes prevention would you like to take advantage of?” with ten mutually nonexclusive answers. Respondents were asked to select “yes” or “no” for each answer choice.

Health status: Respondents were asked about their health status, using the following questions: “Has ever doctor said that you had diabetes?” (If yes, which type of diabetes you had diagnosed: type 1, type 2, gestational diabetes, other types of diabetes, I do not remember), and “Do you have chronic diseases or health problems lasting at least 6 months (yes/no). Moreover, respondents were asked about the history of diabetes in the family, using the question: “Does anyone in your immediate family have diabetes (e.g., children, parents, siblings, grandparents)?” (yes/no).

Sociodemographic characteristics included gender, age, educational level, marital status, having children, place of residence, number of household members, occupational status (active—currently employed or self-employed or passive—unemployed, retired, student), and self-declared financial status.

### Data Analysis

Data were analyzed using SPSS package version 28 (IBM, Armonk, NY, USA). The distribution of categorical variables was presented with frequencies and proportions. Cross-tabulations and chi-square tests were used to compare categorical variables. The statistical significance level was set at *p* < 0.05.

## Results

### Characteristics of the Study Population

This study is based on responses from 1,046 adults in Poland (53.4% were females). Among the respondents, 43.6% had chronic diseases, and 14.1% were diagnosed with diabetes (Table 1). Over one-third of respondents (36.7%) declared a history of diabetes in the immediate family (Table 1).

**TABLE 1:** Characteristics of the study population (N = 1,046) (Warsaw, Poland, 2023).

Variable	Total sample N = 1,046	
	n	%
Gender		
Female	559	53.4
Male	487	46.6
Age (years)		
18–29	179	17.1
30–39	230	22.0
40–49	180	17.2
50–59	122	11.7
60+	335	32.0
Educational level		
Primary	25	2.4
Vocational	102	9.8
Secondary	450	43.0
Higher	469	44.8
Marital status		
Single	280	26.8
Married	552	52.8
Informal relationship	173	16.5
Divorced/widowed	41	3.9
Having children		
Yes	696	66.5
No	350	33.5
Place of residence		
Rural area	378	36.1
City below 20,000 inhabitants	127	12.1
City from 20,000 to 99,999 inhabitants	215	20.6
City from 100,000 to 499,999 inhabitants	191	18.3
City above 500,000 inhabitants	135	12.9
Number of household members		
1	176	16.8
2	369	35.3
3 or more	501	47.9
Occupational status		
Active	596	57.0
Passive	450	43.0
Financial status		
Good	465	44.5
Moderate	371	35.5
Bad	210	20.1
History of diabetes in the family		
Yes	384	36.7
No	662	63.3
Presence of chronic diseases		
Yes	456	43.6
No	590	56.4
Having diabetes diagnosed by a doctor		
Yes	148	14.1
No	898	85.9

### Public Expectations and Needs Related to Type 2 Diabetes Prevention

Most of the respondents (77.3%) declared willingness (definitely yes or rather yes) to take advantage of activities aimed at preventing type 2 diabetes (Table 2). Out of 10 different diabetes prevention measures analyzed in this study, having a blood glucose level measurement performed at a pharmacy (75.4%), medical consultation with a diabetologist (75.1%), family doctor consultation (74.9%), dietary consultation (71.8%), and BMI calculation (70.6%) were the most common type 2 diabetes prevention measures expected by public in Poland (Table 2).

**TABLE 2:** Public expectations and needs related to type 2 diabetes prevention (N = 1,046) (Warsaw, Poland, 2023).

Variable	Total sample N = 1,046	
	n	%
Would you like to take advantage of activities aimed at preventing type 2 diabetes?		
Definitely yes	409	39.1
Rather yes	400	38.2
Rather no	81	7.7
Definitely no	32	3.1
Difficult to tell	124	11.9
Which activities aimed at type 2 diabetes prevention, would you like to take advantage of? – positive answers		
Blood glucose level measurement performed at a pharmacy	789	75.4
Medical consultation with a diabetologist	786	75.1
Family doctor consultation	783	74.9
Dietary consultation	751	71.8
height and weight measurement (BMI calculation)	738	70.6
Sports activities	673	64.3
Advice on physical activity	645	61.7
Consultation with a health educator or nurse	633	60.5
Dietary or culinary workshops	608	58.1
Outdoor events or stands during special events (e.g., “health picnic”)	602	57.6

### Sociodemographic Differences in Public Expectations Towards Type 2 Diabetes Prevention

There were sociodemographic differences in public expectations towards participation in activities aimed at preventing type 2 diabetes (Table 3). Respondents with higher education (84.0%) more often declared interest in type 2 diabetes prevention measures compared to other educational groups (*p* < 0.001). The lowest percentage of respondents who declared willingness to participate in activities aimed at preventing type 2 diabetes was observed among respondents who declared being single (68.2%; <0.001). Respondents with a history of diabetes in the family more often declared willingness to participate in activities aimed at preventing type 2 diabetes compared to those without a history of diabetes in the family (82.8% vs. 74.2%; *p* = 0.001). Moreover, respondents with chronic diseases more often declared interest in type 2 diabetes prevention measures (80.9% vs. 74.6%; *p* = 0.02) compared to healthy individuals (Table 3).

**TABLE 3:** Sociodemographic differences in public expectations towards participation in activities aimed at preventing type 2 diabetes (N = 1,046) (Warsaw, Poland, 2023).

Variable	Would you like to take advantage of activities aimed at preventing type 2 diabetes? – responses definitely yes or rather yes		
	Total sample (N = 1,046)		
	n	%	*p*
Gender			
Female	431	77.1	0.8
Male	378	77.6	
Age (years)			
18–29	138	77.1	0.8
30–39	174	75.7	
40–49	145	80.6	
50–59	96	78.7	
60+	256	76.4	
Educational level			
Primary	14	56.0	**<0.001**
Vocational	73	71.6	
Secondary	328	72.9	
Higher	394	84.0	
Marital status			
Single	191	68.2	**<0.001**
Married	444	80.4	
Informal relationship	142	82.1	
Divorced/widowed	32	78.0	
Having children			
Yes	547	78.6	0.2
No	262	74.9	
Place of residence			
Rural area	302	79.9	0.2
City below 20,000 inhabitants	89	70.1	
City from 20,000 to 99,999 inhabitants	171	79.5	
City from 100,000 to 499,999 inhabitants	145	75.9	
City above 500,000 inhabitants	102	75.6	
Number of household members			
1	129	73.3	0.2
2	294	79.7	
3 or more	386	77.0	
Occupational status			
Active	468	78.5	0.3
Passive	341	75.8	
Financial status			
Good	371	79.8	0.2
Moderate	279	75.2	
Bad	159	75.7	
History of diabetes in the family			
Yes	318	82.8	**0.001**
No	491	74.2	
Presence of chronic diseases			
Yes	369	80.9	**0.02**
No	440	74.6	
Having diabetes diagnosed by a doctor			
Yes	120	81.1	0.2
No	689	76.7	

Bold font was used to mark the results that met the statistical significance criteria (*p* < 0.05).

There were sociodemographic differences in public expectations towards activities aimed at type 2 diabetes prevention (Table 4). Having a family member diagnosed with diabetes had a significant influence on the likelihood of choosing all but one (consultation with a family doctor, *p* = 0.1) preventive services. Marital status was also linked to significant differences in preferences. Single respondents were less likely to opt for all but two (advice on physical activity, *p* = 0.3 and sports activities, *p* = 0.08) preventive actions. People diagnosed with diabetes more often than healthy respondents declared a willingness to participate in 6 out of 10 preventive activities. The self-declared financial status of respondents had no significant influence on their preferences, and the place of residence was linked only with a higher interest in outdoor events or stands during special events (61.1% vs. 48.9% in the biggest cities, *p* = 0.03).

**TABLE 4:** Sociodemographic differences in public expectations towards activities aimed at type 2 diabetes prevention (N = 1,046) (Warsaw, Poland, 2023).

Variable	Dietary consultation			Dietary or culinary workshops			Advice on physical activity			Sports activities			BMI calculation		
	n	%	*p*	n	%	*p*	n	%	*p*	n	%	*p*	n	%	*p*
Gender															
Female	424	75.8	**0.002**	361	64.6	**<0.001**	354	63.3	0.2	375	67.1	**0.04**	406	72.6	0.1
Male	327	67.1		247	50.7		291	59.8		298	61.2		332	68.2	
Age (years)															
18–29	127	70.9	0.9	118	65.9	0.2	117	65.4	0.6	121	67.6	**0.01**	115	64.2	0.2
30–39	166	72.2		136	59.1		148	64.3		161	70.0		167	72.6	
40–49	128	71.1		102	56.7		107	59.4		121	67.2		125	69.4	
50–59	86	70.5		65	53.3		73	59.8		79	64.8		83	68.0	
60+	244	72.8		187	55.8		200	59.7		191	57.0		248	74.0	
Educational level															
Primary	17	68.0	**0.04**	15	60.0	0.4	16	64.0	0.4	13	52.0	**0.01**	15	60.0	0.6
Vocational	62	60.8		52	51.0		55	53.9		53	52.0		69	67.6	
Secondary	321	71.3		260	57.8		277	61.6		286	63.6		319	70.9	
Higher	351	74.8		281	59.9		297	63.3		321	68.4		335	71.4	
Marital status															
Single	180	64.3	**0.01**	145	51.8	**0.02**	160	57.1	0.3	164	58.6	0.08	169	60.4	**<0.001**
Married	413	74.8		325	58.9		349	63.2		361	65.4		416	75.4	
Informal relationship	125	72.3		108	62.4		110	63.6		121	69.9		122	70.5	
Divorced/widowed	33	80.5		30	73.2		26	63.4		27	65.9		31	75.6	
Having children															
Yes	513	73.7	0.05	404	58.0	0.9	436	62.6	0.4	449	64.5	0.9	512	73.6	**0.003**
No	238	68.0		204	58.3		209	59.7		224	64.0		226	64.6	
Place of residence															
Rural area	288	76.2	0.2	228	60.3	0.6	243	64.3	0.4	250	66.1	0.7	272	72.0	0.8
City below 20,000 inhabitants	89	70.1		68	53.5		73	57.5		76	59.8		84	66.1	
City from 20,000 to 99,999 inhabitants	148	68.8		123	57.2		131	60.9		134	62.3		152	70.7	
City from 100,000 to 499,999 inhabitants	134	70.2		107	56.0		122	63.9		125	65.4		136	71.2	
City above 500,000 inhabitants	92	68.1		82	60.7		76	56.3		88	65.2		94	69.6	
Number of household members															
1	121	68.8	0.6	102	58.0	0.8	101	57.4	0.2	108	61.4	0.1	115	65.3	0.3
2	265	71.8		210	56.9		222	60.2		227	61.5		264	71.5	
3 or more	365	72.9		296	59.1		322	64.3		338	67.5		359	71.7	
Occupational status															
Active	421	70.6	0.3	347	58.2	0.9	367	61.6	0.9	410	68.8	**<0.001**	416	69.8	0.5
Passive	330	73.3		261	58.0		278	61.8		263	58.4		322	71.6	
Financial status															
Good	340	73.1	0.7	275	59.1	0.5	293	63.0	0.7	314	67.5	0.2	332	71.4	0.9
Moderate	264	71.2		219	59.0		224	60.4		229	61.7		260	70.1	
Bad	147	70.0		114	54.3		128	61.0		130	61.9		146	69.5	
History of diabetes in the family															
Yes	295	76.8	**0.006**	252	65.6	**<0.001**	255	66.4	**0.02**	276	71.9	**<0.001**	289	75.3	**0.01**
No	456	68.9		356	53.8		390	58.9		397	60.0		449	67.8	
Presence of chronic diseases															
Yes	346	75.9	**0.01**	275	60.3	0.2	295	64.7	0.08	295	64.7	0.8	329	72.1	0.3
No	405	68.6		333	56.4		350	59.3		378	64.1		409	69.3	
Having diabetes diagnosed by a doctor															
Yes	118	79.7	**0.02**	98	66.2	**0.03**	107	72.3	**0.01**	100	67.6	0.4	117	79.1	**0.01**
No	633	70.5		510	56.8		538	59.9		573	63.8		621	69.2	
(Continued on following page)																

Bold font was used to mark the results that met the statistical significance criteria (*p* < 0.05).

### Factors Associated With Public Expectations Towards Participation in Activities Aimed at Preventing Type 2 Diabetes

In multivariable logistic regression, having higher education (OR: 3.83, 95% CI: 1.64–8.94, *p* = 0.002), having chronic diseases (OR: 1.36, 95% CI: 1.01–1.85, *p* = 0.04), and history of diabetes in the family (OR: 1.67, 95% CI: 1.21–2.30, *p* = 0.002) were significantly associated with higher interest in participation in activities aimed at preventing type 2 diabetes (Table 5). A separated analysis was performed for participants without diagnosis of diabetes (*n* = 898), also confirmed that having higher education (OR: 3.84, 95%CI: 1.53–9.68, *p* = 0.004), history of diabetes in the family (OR: 1.86, 95%CI: 1.30–2.65, *p* < 0.001) and presence of chronic diseases (OR: 1.64, 95%CI: 1.16–2.30, *p* = 0.01) were significantly associated with higher interest in participation in activities aimed at preventing type 2 diabetes (Table 6).

**TABLE 5:** Factors associated with public expectations towards participation in activities aimed at preventing type 2 diabetes (N = 1,046) (Warsaw, Poland, 2023).

Variable	Factors associated with public expectations towards participation in activities aimed at preventing type 2 diabetes (N = 1,046)					
	Univariable logistic regression			Multivariable logistic regression		
	OR	95%CI	*p*	OR	95% CI	*p*
Gender						
Female	0.97	0.73–1.30	0.8			
Male		Reference				
Age (years)						
18–29	1.04	0.68–1.60	0.9			
30–39	0.96	0.65–1.42	0.8			
40–49	1.28	0.82–2.00	0.3			
50–59	1.14	0.69–1.88	0.6			
60+		Reference				
Educational level						
Primary		Reference			Reference	
Vocational	1.98	0.80–4.86	0.1	1.81	0.72–4.56	0.2
Secondary	2.11	0.93–4.78	0.07	2.00	0.87–4.62	0.09
Higher	4.13	1.81–9.44	**<0.001**	3.83	1.64–8.94	**0.002**
Marital status						
Married	1.45	1.09–1.94	**0.01**	1.31	0.98–1.77	0.08
Unmarried		Reference			Reference	
Having children						
Yes	1.23	0.91–1.67	0.2			
No		Reference				
Place of residence						
Rural area	1.29	0.81–2.05	0.3			
City below 20,000 inhabitants	0.76	0.44–1.31	0.3			
City from 20,000 to 99,999 inhabitants	1.26	0.75–2.10	0.4			
City from 100,000 to 499,999 inhabitants	1.02	0.61–1.71	0.9			
City above 500,000 inhabitants		Reference				
Number of household members						
1	0.82	0.55–1.21	0.3			
2	1.17	0.84–1.62	0.4			
3 or more		Reference				
Occupational status						
Active	1.17	0.87–1.56	0.3			
Passive		Reference				
Financial status						
Good	1.27	0.86–1.87	0.2			
Moderate	0.97	0.66–1.44	0.9			
Bad		Reference				
History of diabetes in the family						
Yes	1.68	1.22–2.30	**0.001**	1.67	1.21–2.30	**0.002**
No		Reference			Reference	
Presence of chronic diseases						
Yes	1.45	1.07–1.95	**0.02**	1.36	1.01–1.85	**0.04**
No		Reference			Reference	
Having diabetes diagnosed by a doctor						
Yes	1.30	0.84–2.02	0.2			
No		Reference				

Bold font was used to mark the results that met the statistical significance criteria (*p* < 0.05).

**TABLE 6:** Factors associated with public expectations towards participation in activities aimed at preventing type 2 diabetes among those without diabetes (N = 898) (Warsaw, Poland, 2023).

Variable	Factors associated with public expectations towards participation in activities aimed at preventing type 2 diabetes among those without diabetes (N = 898)						
	Univariable logistic regression				Multivariable logistic regression		
	OR	95%CI	*p*		OR	95% CI	*p*
Gender							
Female	1.02	0.75–1.40	0.9				
Male		Reference					
Age (years)							
18–29	1.10	0.69–1.75	0.7				
30–39	0.89	0.58–1.37	0.6				
40–49	1.18	0.73–1.91	0.6				
50–59	1.04	0.61–1.76	0.9				
60+		Reference					
Educational level							
Primary		Reference				Reference	
Vocational	2.39	0.90–6.34	0.08		2.20	0.80–6.04	0.1
Secondary	2.48	1.02–6.01	**0.04**		2.32	0.93–5.77	0.07
Higher	4.22	1.73–10.31	**0.002**		3.84	1.53–9.68	**0.004**
Marital status							
Married	1.42	1.04–1.93	**0.03**	1.26		0.91–1.74	0.2
Unmarried		Reference				Reference	
Having children							
Yes	1.14	0.83–1.57	0.4				
No		Reference					
Place of residence							
Rural area	1.36	0.82–2.26	0.2				
City below 20,000 inhabitants	0.71	0.39–1.27	0.2				
City from 20,000 to 99,999 inhabitants	1.23	0.71–2.14	0.5				
City from 100,000 to 499,999 inhabitants	0.96	0.55–1.67	0.9				
City above 500,000 inhabitants		Reference					
Number of household members							
1	0.82	0.54–1.25	0.4				
2	1.17	0.82–1.66	0.4				
3 or more		Reference					
Occupational status							
Active	1.10	0.80–1.51	0.6				
Passive		Reference					
Financial status							
Good	1.11	0.73–1.68	0.6				
Moderate	0.88	0.57–1.34	0.5				
Bad		Reference					
History of diabetes in the family							
Yes	1.83	1.29–2.59	**<0.001**	1.86		1.30–2.65	**<0.001**
No		Reference				Reference	
Presence of chronic diseases							
Yes	1.74	1.25–2.44	**0.001**	1.64		1.16–2.30	**0.01**
No		Reference				Reference	

Bold font was used to mark the results that met the statistical significance criteria (*p* < 0.05).

## Discussion

To the authors’ best knowledge, this is the first study on the expectations and needs relating to type 2 diabetes prevention and on factors associated with willingness to participate in activities aimed at type 2 diabetes prevention among adults in Poland. Most of the respondents declared interest in diabetes-preventing activities was high. Out of Educational level, history of diabetes in the family and presence of chronic diseases were significantly associated with expectations towards participation in activities aimed at preventing type 2 diabetes among those without diabetes, both among all participants as well as those without diabetes.

Those results correspond to the previously published data [27] on the awareness of diabetes in Polish adults. As it was recently confirmed by Sękowski et al. [11], knowledge of diabetes in Poland is strongly related to the patient’s level of education. Rising awareness of diabetes among Polish adults, its risk factors and symptoms results in higher readiness to participate in diabetes prevention actions. However, an educational gradient of this attitude may result in greater health inequalities [28], as only a part of the population is ready to take advantage of such preventive measures. This is especially of consideration in the case of Poland, where a substantial part of the population had never undergone a blood glucose test [29], and the number of undiagnosed patients with type 2 diabetes is estimated to be up to 1 million [30].

Most of the respondents declared willingness to participate in diabetes prevention activities based on healthcare services such as blood sugar tests, consultations with specialists (diabetologists) and family doctors. Lifestyle-related interventions were the least chosen. Those preferences towards medical services and, in consequence, secondary prevention may be due to two reasons.

The Polish health system is considered to have problems in meeting the health needs of the society [31, 32]. Therefore, patients may prefer actions that provide them with the opportunities to meet the demand for health services. This seems especially true for the oldest patients (60+ age group), who most often choose the medical interventions through consultations with a family doctor or a diabetologist (89.5% and 80%, respectively). The inadequate availability of medical services is also perceived as a problem by doctors who provide such services to senior patients [33].

On the other hand, the knowledge of lifestyle-related risk factors for diabetes remains inadequate in Poland [12]. This may result in higher demand for services that meet the social notion of effective diabetes prevention.

Numerous studies have determined the influence of physical activity and exercise on health outcomes. The research indicates that physical activity increases the quality of life and lowers death rates with little to no safety issues [34]. A recent study by Biernat et al. [35] revealed low declared physical activity levels among Poland’s adults. It showed that the likelihood of participation in physical activities depends among other things, on age and education level. Those findings correspond with the results of this study in which sports activities, as a form of diabetes prevention, were chosen mostly by younger participants (70% in the 30–39 age group) with higher education (68.4%) and active occupational status (68.8%). The latter correlates with the findings of Meyer et al. [36], who showed that higher socio-economic status was associated with involvement in all intensity levels of physical activity.

The diet is a second lifestyle factor considered key in diabetes prevention and mitigation of its complications [37]. Some studies show that due to dietary intake, never-married men in Poland manifest, on average, higher systolic and diastolic blood pressure [38] – as risk factors for diabetes [39] and common comorbidities [40]. Despite those circumstances, single respondents were less willing to participate in all diet-related activities (culinary workshops, dietary consultations). Moreover, being single was also negatively correlated with lower preferences towards all consultations with healthcare professionals (family doctors, diabetologists, nurses and educators). Contrasted with, on average, lower quality of life and perceived health condition observed among non-married adults in Poland [41], the attitude of single adults towards preventive health services identified in this study poses a serious challenge.

The results of this study also demonstrated that having a family member with diabetes increases the willingness to participate in all but one (consultation with family doctor) activities. We can hypnotically assume that this is because diabetes has a particular characteristic in that it is a chronic disease that typically manifests in older age, and the patient frequently needs family support and involvement in disease management. Therefore, the patient’s family may have a higher-than-average understanding of this disease (its risk factors and its prevention), as reported by Sękowski et al. [12]. These may complement proper diabetic education that should be offered as part of a public health intervention on diabetes but should not be used in its place. This study has practical implications for the implementation of a community pharmacist-physician collaborative working model in Poland. This study revealed that there is a need to strengthen the role of pharmacists in interprofessional care. Pharmacists should be encouraged to perform drug reviews and report drug interactions and polypharmacy. Pharmacist-physician collaborative working should be included in postgraduate training programs. Both groups should improve communication skills and learn how to build proper relationships based on respect and trust. There is also a need to promote the role of the pharmacist in the healthcare system - no longer as a distributor of drugs, but as an advisor and consultant in matters related to pharmacotherapy [18]. Public authorities should remove barriers to pharmacist-physician collaborative working. Pharmacists should gain access to basic electronic health records that are necessary for pharmaceutical care [16, 42]. Moreover, the development of clear guidelines on what information should be recorded in the patients’ records from the point of view of optimizing pharmacotherapy will also contribute to better cooperation between pharmacists and physicians.

### Practical Implications

Numerous practical ramifications for public health actions in Poland can be drawn from this study. It shows a high level of interest in participating in diabetes prevention activities among adults in Poland. This interest is, however, directed towards medical services, while willingness to participate in lifestyle interventions is lower.

This finding emphasizes a need for improved education on diabetes, with special consideration for its risk factors and prevention methods. It is important to pay close attention to communication addressed to individuals with lower education, as that group was identified as less likely to participate in any diabetes prevention activities. Single males, especially those not active in the labour market, should be approached with targeted actions focusing on lifestyle risk factors and prevention methods of diabetes.

Moreover, this study provides evidence of inadequate education of patients diagnosed with diabetes in Poland and their low motivation to participate in activities that could prevent or delay complications of their disease. The results of this study also highlight the beneficial effect that having a family member with diabetes has on the degree of diabetes awareness among other family members.

The findings of this study can have implications for other countries, particularly those that are facing similar epidemics and economic challenges, like the former Eastern block countries. Nonetheless, it’s important to consider that social and cultural differences may also play a role.

### Limitations

This research has several limitations. The CAWI research approach was used to conduct the study, which forgoes direct interaction between the interviewer and the respondent (for example, the ability to judge the respondents’ abilities and capacity to comprehend the questions posed). The study’s questionnaire was restricted to the most common activities offered in the Polish health system. Due to the study’s design, medical records were not validated, and participants self-reported health information and information on their family health history. Nevertheless, this is the most thorough and recent survey on the general public’s attitude towards diabetes prevention activities conducted among adults in Poland.

### Conclusion

The findings of the study were unequivocal and demonstrated a remarkable level of interest among Polish adults in activities aimed at preventing diabetes. Notably, the education level of respondents emerged as a crucial and statistically significant factor linked to their willingness to participate in such activities. Additionally, the study revealed a preference for medical interventions over lifestyle-oriented interventions, with only younger, better-educated, and working respondents showing a greater inclination towards the latter.

These results underscore the importance of comprehensive diabetes education, particularly in relation to lifestyle-related risk factors and prevention methods.
